# Acute Retinal Necrosis: Clinical Features, Diagnostic Pitfalls, Treatment, and Outcome of an Insidious Disease in Children. Case Report and Review of the Literature

**DOI:** 10.3389/fped.2022.854325

**Published:** 2022-04-01

**Authors:** Chiara Mapelli, Paolo Milella, Caterina Donà, Marco Nassisi, Silvia Osnaghi, Francesco Viola, Carlo Agostoni, Francesca Minoia, Giovanni Filocamo

**Affiliations:** ^1^Fondazione IRCCS Ca' Granda Ospedale Maggiore Policlinico di Milano, Milan, Italy; ^2^Department of Clinical Sciences and Community Health, University of Milan, Milan, Italy

**Keywords:** herpes virus, acute retinal necrosis (ARN), treatment, pediatric, retina

## Abstract

**Objective:**

This study aims to explore clinical features, diagnostic work-up, treatment, and outcomes of pediatric patients with acute retinal necrosis (ARN), and to propose a standardized management of this condition in childhood.

**Methods:**

Clinical manifestations, diagnostic work-up, and treatment of three pediatric cases with ARN were analyzed. Furthermore, a review of the literature was performed from January 1990 to November 2021, focused on 1) clinical presentation; 2) differential diagnosis, including both infectious and non-infectious conditions; 3) key role of diagnostic techniques; and 4) currently available treatments.

**Results:**

Data from 72 children with ARN (69 from literature and 3 from our center) were analyzed. The most frequent presenting symptoms were red eye resistant to topical treatment (57%) and altered vision (58%), 25 patients had bilateral involvement. In 30% a known history of herpetic infection was reported. PCR testing on anterior chamber and/or vitreous sampling was performed in 46 cases (64%) and was diagnostic in 88% of them, with herpes simplex virus (HSV) 2 being the most frequently identified pathogen (57%). All patients underwent systemic antiviral therapy (16% only oral); adjunctive intravitreal injections were performed in 21% of them.

**Conclusions:**

ARN is a rare but severe ocular infection presenting as a panuveitis with occlusive retinal vasculitis and peripheral retinal necrosis. Varicella-zoster virus and HSV 1–2 are most frequently implicated. Due to a high incidence of rhegmatogenous retinal detachment and optic atrophy, ARN has a poor prognosis with a potentially severe impact on visual function. Although a prompt recognition is crucial to prevent complications, ARN diagnosis in children is still challenging.

## Introduction

Acute retinal necrosis (ARN) is a panuveitis characterized by rapidly progressive circumferential or confluent retinitis associated with various degrees of vitritis and vasculitis, often occlusive (1). It is a rare condition that can affect any gender and age, with an estimated annual incidence of about 0.5–0.63 new cases per million in the general population (1). In the pediatric population, ARN is even more uncommon, with an exiguous number of cases described in literature and no guidelines for work-up and treatment.

ARN is usually monolateral and symptoms are aspecific as they mainly include red eye with mild eye pain and sudden decreased vision. The pathogenic mechanism is related to an intraocular herpetic infection from varicella-zoster virus (VZV), herpes simplex virus 1–2 (HSV 1–2), or, more rarely, Epstein–Barr virus (EBV) or cytomegalovirus (CMV) (2).

Besides well-known acquired or inherited defects of cellular and innate immunity, which increase the risk of recurrent and invasive herpetic infections, ARN has been reported also in otherwise healthy children, suggesting a potential predisposing role of selective immunological defects in the response to herpesviruses (3–5). Due to the rarity of this condition and the lack of specific symptoms, recognizing ARN in children may be challenging. This study aims to describe clinical features, diagnostic work-up, treatment, and outcome of pediatric patients with ARN through a case series and a review of the literature.

## Methods

Medical records, including clinical and ophthalmological features, laboratory and imaging results, and treatment of three pediatric cases with ARN treated at our center were reviewed. Furthermore, a review of the literature published in English on PubMed between January 1990 and November 2021 was performed. Keywords for literature research were “acute retinal necrosis” AND “childhood”; further studies were identified through manual search of the reference lists of the selected papers. Literature review was focused on 1) clinical presentation; 2) differential diagnosis, including both infectious and non-infectious conditions; 3) key role of diagnostic techniques, as molecular detection on intraocular fluids; and 4) currently available treatments and their optimal duration to prevent recurrences and long-term sequelae.

A mixed effect logistic regression with available data was performed to identify possible prognostic factors for visual acuity improvement (at least 0.1 LogMAR).

## Case Presentation

### Case 1

**Figure 1:**
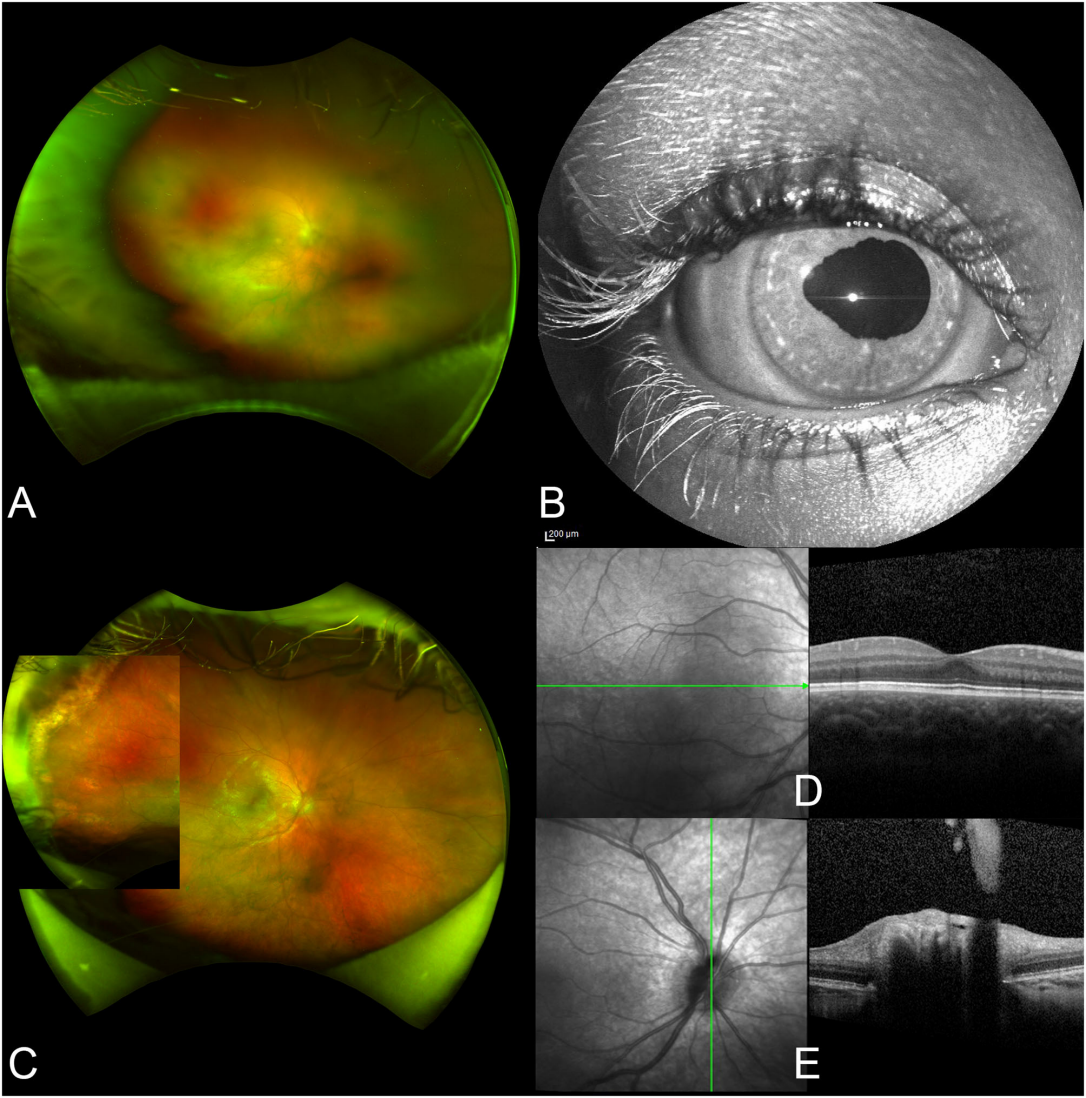
Ophthalmic images from case 1. At presentation, the vitritis does not allow a detailed visualization of the retina **(A)**. Anterior segment examination shows an irregular shape of the pupil **(B)**. After treatment, the vitritis resolved and chorioretinal scars are seen in the far temporal periphery of the retina **(C)**. Optical coherence tomography shows a physiologic macular profile **(D)**, while there is a residual peripapillary vitreous opacification in the optic nerve scan **(E)**.

A 5-year-old boy with a 1-week history of mild persistent hyperemia and irregular pupil shape in the right eye (OD) was sent by his pediatrician for ophthalmic evaluation. His medical history was remarkable for an HSV-1-related necrotic-hemorrhagic encephalitis that occurred at the age of 18 months, which was successfully treated with intravenous acyclovir. At that time, no ocular involvement was reported, immunological analyses resulted within normal ranges for age, and next-generation sequencing (NGS) panel for primary immunodeficiencies was negative. At presentation, best-corrected visual acuity (BCVA) was 20/20 in the left eye (OS) and hand motion in OD. Slit-lamp and fundus examinations were unremarkable in OS, while OD showed a granulomatous anterior uveitis with posterior synechiae, and a severe vitritis, which did not allow a proper visualization of the retina (Figures 1A,B). With the clinical suspect of ARN, the patient was hospitalized and promptly treated with intravenous acyclovir, topical dexamethasone, and tropicamide. After 5 days, oral prednisone (1 mg/kg/die) was introduced to reduce the vitritis. Systemic work-up showed positive IgG for HSV-1, CMV, and VZV, while HSV-2, Treponema Pallidum, Tuberculosis, and Borrelia were negative. Aqueous sampling protein chain reaction (PCR) analysis confirmed the presence of HSV-1 DNA, although it was negative on serum. At 6 weeks, BCVA in OD improved to 20/25, anterior chamber (AC) inflammation and vitritis resolved, and fundus examination showed peripheral pigmented scars with no evidence of active retinitis. The patient was therefore discharged with oral acyclovir and tapering prednisone. Oral corticosteroid was administered for a total of 6 months, while oral acyclovir was continued for 24 months as prophylaxis. Six weeks after stopping the antiviral treatment, the patient had a recurrence of a cutaneous herpetic infection, leading to the re-introduction of the prophylactic oral acyclovir. At the last follow-up visit, 30 months after presentation, BCVA was 20/32 in OD due to subcapsular cataract; no signs of ocular inflammation were detected (Figures 1C–E). Considering the increasing discovery of new inborn error of immunity related to invasive or recurrent herpetic infection, whole exome sequencing of patient and parents was performed, without detection of pathogenetic variants associated to the phenotype.

### Case 2

**Figure 2:**
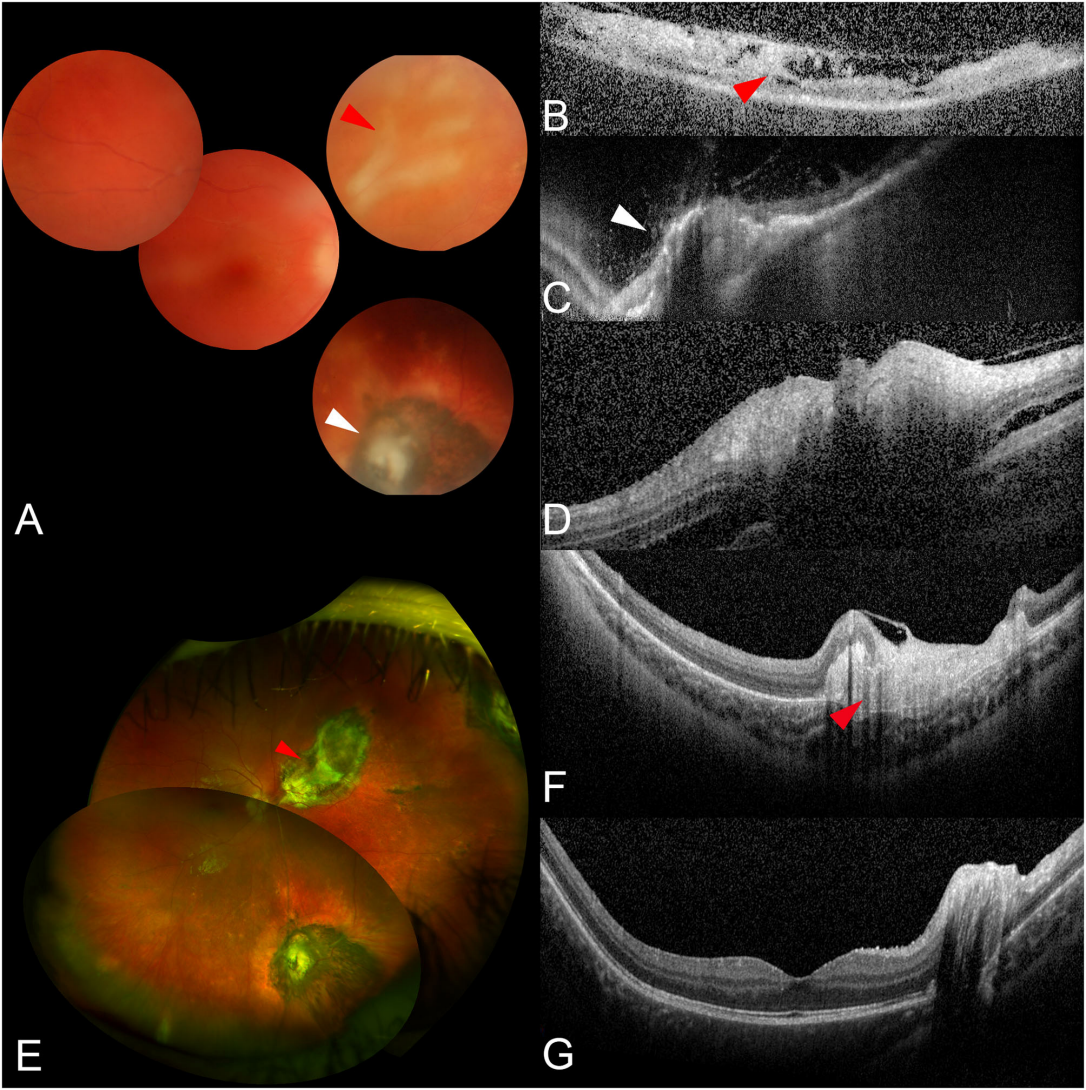
Ophthalmic images from case 2. At presentation, despite the vitritis, it is possible to distinguish areas of retinitis (**A**, red arrowhead) and a pigmented chorioretinal scar (**A**, white arrowhead) through fundus examination **(A)**. Optical coherence tomography (OCT) scans confirm the presence of inflammatory hyper-reflective material and retinal layer disorganization on the areas of retinitis (**B**, red arrowhead), and retinal atrophy with pigment clumping on the chorioretinal scar (**C**, white arrowhead). Finally, an OCT scan through the optic disc shows a swollen nerve head with peripapillary subretinal fluid **(D)**. After treatment, the vitritis resolved and a large chorioretinal atrophy is now visible in correspondence of the previous retinitis in both fundus examination (**E**, red arrowhead) and OCT (**F**, red arrowhead). OCT also shows a physiologic macular profile **(G)**.

A 7-year-old girl was referred to our center for a 1-week history of blurred vision in her right eye. She had a previous episode of anterior uveitis of unknown etiology at 6 months of age and her general medical history was remarkable for recurrent aphthosis and irritant dermatitis. The ophthalmic examination was unremarkable for OS, while OD had a BCVA of hand motion and showed a moderate AC inflammation with posterior synechiae, and vitritis. Fundus examination was arduous but showed optic disc swelling, a partially fibrotic and elevated pigmented chorio-retinal scar in the inferior periphery, and an area of active retinitis—confirmed by OCT—in the nasal quadrant (Figures 2A–D). Intravenous acyclovir was promptly initiated. Serum HSV-1-specific antibody testing was positive for IgG and negative for IgM and serum HSV DNA was negative. However, HSV-1 DNA was detected in the aqueous humor through PCR. A complete uveitis work-up resulted otherwise normal. After 2 weeks, BCVA improved to 20/63, vitritis partially cleared, retinitis was healing, but optic disc swelling was still present; the patient was dismissed with oral acyclovir, prednisone, and topical drops of dexamethasone and tropicamide. Antiviral therapy was discontinued after 6 months. At last follow-up visit (54 months), BCVA was 20/20 in OD and funduscopy showed no evidence of active retinitis (Figures 2E–G).

### Case 3

A 6-year-old boy was referred to our center in 2005 for bilateral panuveitis and retinitis. His medical history was remarkable for a recurrent herpetic cutaneous infection at his feet from 9 months of age. Even though the treatment for ARN was promptly initiated, in 2007 he developed a retinal detachment (RD) in OS, resulting in complete abolition of visual function (no light perception) despite surgical intervention with vitrectomy and scleral buckling. Right eye maintained a BCVA of 20/20, with peripheral retinal scars. A prophylactic treatment with oral acyclovir was sustained and regular follow-ups were performed. At the age of 22, he accessed our urgency care center for blurred vision in OD. Indeed, his BCVA was 20/25 and ophthalmic assessment revealed mild anterior uveitis, vitritis, retinal vasculitis, and an area of active retinitis adjacent to a peripheral chorioretinal pigmented scar in the nasal quadrant. A diagnostic AC paracentesis was positive for HSV-2-DNA and endovenous acyclovir was started; however, since ARN recurred despite prophylactic treatment, a pharmacoresistance to acyclovir was suspected. Following specialistic infectious disease consultation, acyclovir was switched to endovenous foscarnet for 21 days. Oral prednisone 25 mg daily was also introduced after 3 days. After 2 weeks, vitritis and retinitis progressively resolved. The patient was then discharged with oral famciclovir, prednisone 5 mg/die, and topical steroid drops, slowly tapered. At last follow-up visit, 8 months later, BCVA improved to 20/20 in OD, ocular inflammation regressed and there was no evidence of active retinitis. Prophylactic therapy with famciclovir was continued.

## Literature Review–Based Case Series

**Figure 3:**
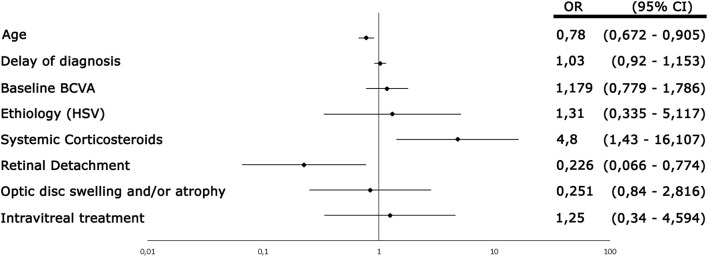
Forest plot showing the result of the regression analysis performed on the published data. Age, retinal detachment, and use of corticosteroids were the only parameters associated to best-corrected visual acuity (BCVA) improvement. HSV: herpes simplex virus, OR: odds ratio; CI: confidence interval.

Between January 1990 and November 2021, 69 children with ARN were reported in literature and data from 72 patients, including the 3 children treated at our center, were analyzed. Main demographic, clinical features, laboratory findings treatment, and outcome of this review-based case series are reported in Table 1, while a detailed report of the 72 cases collected is available in Supplementary Table 1. The most frequent presenting symptoms were red eye resistant to topical treatment (57%) and altered vision (58%), and 25 patients had bilateral involvement. In 30% of patients, a known history of herpetic infection was reported. PCR testing on AC and/or vitreous sampling was performed in 46 cases (64%) and was diagnostic in 88% of them, with HSV-2 being the most frequently identified pathogen (57%). Acyclovir was the most used antiviral drug with a variable duration of treatment between 6 weeks and 16 years. Therapeutic phase was followed by an antiviral prophylaxis in 39 cases (54%). Indeed, recurrences happened in 8 cases with a median interval of 9.5 years. In 4 of these cases, the recurrence involved the same eye, while in 4 patients it developed in the fellow eye. Vision prognosis was poor, with only 52% of eyes retaining a vision of 20/200 or better at the last available follow-up (data available for 51 patients). The most reported complications were RD (34% of cases) and optic atrophy (6%). RD typically presents 1–3 months after the onset of symptoms, even if delayed occurrence has also been reported (up to 18 months). Analyzing data from our literature review, we found an association between BCVA improvement and age [odds ratio (OR) 0.78, 95% confidence interval (CI) 0.672–0.905, *p*-value 0.001], retinal detachment (OR 0.226, CI 0.066–0.774, *p*-value 0.018), and use of systemic corticosteroids (OR 4.8, CI 1.43–16.107, *p*-value 0.011). All other considered parameters were not significantly associated (Figure 3).

**Table 1:** Demographic, clinical features, diagnostic work-up, treatment, and outcome of the 72 pediatric patients with ARN collected by literature review and analysis of our center data.

	**72 pediatric patients with ARN** **(97 eyes)**
Median age at first episode (IQR), years	9.3 (4–13)
Female (F)/Male (M)	27 F (37.5%)/45 M (62.5%)
Previous known herpetic infection, *n* patients	22 (30%)
Median follow-up, months	23.5 (data available for 57 eyes)
Diagnostic delay (excluding incidental finding), mean ± SD, days	8.19 ± 6.7 (data available for 36 eyes)
Etiology, *n* patients	
HSV-1	8 (11%)
HSV-2	41 (57%)
VZV	7 (10%)
Others	3 (4%)
Undetermined	13 (18%)
Clinical features at presentation	
Bilateral involvement, *n* patients	25 (35%)
BCVA^*^	Data available for 63 eyes of 52 patients
Baseline BCVA, mean ± SD, LogMar	1.08 ± 0.9 (20/250 Snellen equivalent)
Eyes with LogMar ≥1 or light perception, *n* eyes (*n* patients)	37 (34)
Symptoms, *n* patients	Data available for 53 patients
Eye redness	30 (57%)
Eye pain	10 (19%)
Anisocoria	4 (7%)
Altered vision	31 (58%)
Photophobia	4 (7%)
Ophthalmological evaluation, *n* eyes	Data available for all eyes
Prior chorioretinal scar	9 (9%)
Exudative retinal detachment	6 (6%)
Optic disc swelling	23 (24%)
Occlusive vasculitis	19 (19%)
Diagnostic work-up with PCR, *n* patients	Data available for all eyes
AC	33 (45%)
Vitreous	16 (22%)
Both	3 (4%)
iosPCR positive/iosPCR performed	43/49
Antiviral therapy^‡^	Data available for 67 patients
Oral only	11 (16%)
Endovenous induction then oral therapy	56 (84%)
Adjunctive intravitreal antiviral treatment	14 (21%)
Systemic corticosteroid treatment	34 (47%)
Median duration of antiviral prophylaxis, months	22 (data available for 39 patients)
Outcome	
Final BCVA* (LogMar)	Data available for 63 eyes of 51 patients
Final BCVA, mean ± SD, LogMar	1.03 ± 1.15 (20/250 Snellen equivalent)
Eyes with LogMar ≥1 or light/no light perception, *n* eyes (*n* patients)	33
Recurrences	4 (5.5%)
Delayed onset bilateral ARN	4 (5.5%)
Median time to recurrences, years	9.5
Long-term ocular sequelae, *n* eyes	
Retinal detachment	33 (34%)
Optic atrophy	6 (6%)

*n, number; BCVA, best-corrected visual acuity; iosPCR, intra-ocular sampling protein chain reaction; AC, anterior chamber.*

* *For low visual acuities, the following equivalences were used: count fingers = 2 LogMar; hand motion = 3 LogMar; light perception was excluded from calculations (6).*

‡ *One patient had two episodes and was treated with oral therapy in one and with endovenous induction therapy in the other*.

## Discussion

Diagnosis of ARN is based on the identification of the typical ophthalmological features and requires a high suspicion. It is critical to differentiate ARN from other infectious uveitis, especially in immunocompromised patients, and from other non-infectious inflammatory conditions from the very early stage of disease. When ARN is suspected, treatment should be started promptly, even before the results of the intraocular samples analysis (7–9). This prevents further extension of the retinitis and severe ocular complications (10, 11), as well as secondary involvement of the fellow eye or of central nervous system (CNS) (12, 13). Herpes viruses are responsible for ARN; specifically, HSV-2 is the most frequent in young patients (<25 years), HSV-1 during young adulthood (>25 years), and VZV in the elderly (7, 14). Our review confirms this pattern, with HSV-2 identified in 41 cases (57%), followed by HSV-1 in 8 (11%) and VZV in 7 (10%); EBV and CMV in 2 and 1 cases, respectively.

It has been proposed that ARN in children may represent the reactivation of a perinatal infection from HSV-2 rather than a primary disease (15). One hypothesis is that herpes viruses may be latent in cranial ganglia and subsequently migrate in the retina through axons; alternatively, ARN may be a recurrence of a previous undiagnosed intraocular infection. Interestingly, 9 out of the 72 cases reviewed presented pigmented chorioretinal scars at diagnosis, which supports the second hypothesis (Table 1 and Supplementary Table 1) (15–17). Neonatal HSV infection is common and has a broad spectrum of clinical manifestations. Previous, concomitant, or subsequent CNS involvement has been widely reported in patients with unilateral and bilateral ARN, with a prevalence that is inversely related to age (18–20). Latency period can be very long, up to several years (16). Interestingly, an association between ARN and recent trauma and intracranial surgery has been reported, suggesting it could be a triggering mechanism for the recrudescence of a latent infection (21, 22). Among the reviewed cases indeed, 22 patients (30%) had a known history of previous herpetic infection, 14 of which involved CNS.

The inflammatory response to the viral infection has a key role in the pathogenesis of ARN and the host immune state may be crucial in the severity and evolution of the disease. Acquired or inborn errors of cellular and innate immunity are well-known risk factors for invasive and recurrent viral infections, including CNS and ocular involvement (3, 4). These conditions include, but are not limited to, deficiencies of natural-killer (NK) and/or T cells (i.e., severe combined immunodeficiency, GATA2 deficiency, Wiskott–Aldrich syndrome, DOCK2 and DOCK8 deficiency) and disorders of innate immunity (i.e., deficiency of INFγ-receptor, TYK2, and NEMO or gain-of-function mutations of *STAT1*). However, severe herpetic infections, including HSV encephalitis (HSE) and ARN, in otherwise healthy children, are not uncommon. The dichotomy between the high frequency of latent herpetic infection and the rarity of ARN suggests a possible role of a genetically determined susceptibility of the host (23). In recent years, an increasing number of genes, mainly involving the TLR3 pathway genes (*TLR3, UNC93B1, TRIF, TRAF3, TBK1*, and *IRF3*), have been related to a CNS-selective defect in immune response to HSV1-2 and VZV, leading to severe HSV encephalitis (HSE) and recurrent ophthalmic zoster (5). Due to the relevance of CNS latency of HSV1-2 and VZV infection in the pathogenesis of ARN, widening the immune-genetic evaluation of ARN patients could improve the management of this rare condition and identify patients at higher risk of severe, complicated, or recurrent disease.

Clinical presentation of ARN can be subtle in pediatric patients: early symptoms overlap with those of a common conjunctivitis and may be underestimated, delaying specialist consultation. Persistent red eye resistant to topical treatment, altered vision, and ocular discomfort are the most common findings. Older children may complain about blurred vision, while pupil abnormalities (usually caused by posterior synechiae) may be noted by parents in pre-verbal babies. In our review-based case series, in 10% of patients the diagnosis was incidental. The reported clinical pictures in the pediatric population were consistent with the typical presentation of ARN. Patients showed AC and/or vitreous inflammation with progressive peripheral areas of necrotizing retinitis that usually appears as confluent multifocal patches or yellow-white infiltrates in the periphery. In addition, occlusive vasculitis was reported in 19% of cases and disc swelling was present in 23% of eyes. In severe cases of ARN, an exudative RD could be observed early in the disease course, in conjunction with the active inflammation.

Diagnosis of ARN is usually based on clinical presentation; nevertheless, the viral etiology can be confirmed by different techniques, including serological analysis of serum/intraocular fluid (Goldman–Witmer coefficient) or viral cultures. Recently, intraocular fluid sampling for PCR analysis has assumed greater importance in confirming the diagnosis and guiding treatment (7). In the reviewed case series, no complications were reported from intraocular procedures. According to the American Academy of Ophthalmology (AAO) guidelines, both AC and vitreous sampling are sensitive and specific; however, aqueous sampling may be safer and, in our opinion, should be considered when suspecting ARN in pediatric cases (1). Of note, when ARN is suspected, antiviral treatment should never be delayed while awaiting aqueous tap as it does not affect the PCR outcome. Indeed, as the viral load follows three phases (a plateau period, a logarithmic decrease, and a negativization phase), the mean time required for it to be below the detection threshold is estimated to be 51 days or longer, even under treatment (24, 25).

OCT has been reported to be a useful tool for the detection of retinal necrosis (26), showing full-thickness hyper-reflectivity and disruption of the retinal layers in the involved area. Furthermore, some OCT features may be effective in differentiating ARN from other forms of infectious retinitis (i.e., toxoplasma) (27). Despite that, none of the authors reported the use of OCT in the reviewed pediatric cases. In our patients, OCT was important not only to confirm the presence of active retinitis (case 2, Figure 2) but also to monitor the evolution of the retinal lesions and to identify possible complications. Of course, OCT has also several limitations as good quality scans can be difficult to obtain in very young children (a good compliance is required) as well as in the context of vitritis. Nevertheless, we advise its use as a non-invasive complementary tool to clinical examination for diagnosis and follow-up.

According to AAO guidelines, treatment regimen for ARN involves an initial 7–10-day course of IV acyclovir (10 mg/kg every 8 h or 1,500 mg/m^2^ per day divided into 3 doses) followed by oral acyclovir. Since the advent of newer oral antivirals with greater bioavailability, multiple studies have reported successful outcomes using oral antiviral treatment (with or without intravitreal therapy) without concomitant intravenous treatment (1). There are no specific therapeutic guidelines for ARN in children. Most of the authors reported use of intravenous acyclovir at a dosage of 30 mg/kg/die; however, given the severity of the disease and the potential CNS co-involvement, a higher dosage up to 60 mg/kg/die could be considered especially in younger children. Systemic antiviral therapy has been proven to reduce the risk of developing bilateral ARN in patients with unilateral disease up to 2 years after initial presentation, so it should always be performed until ocular and systemic inflammation were subsided (12). The duration of oral treatment is typically continued for 6 weeks up to 1 year following initial infection (28). There are no guidelines to suggest how long ARN patients should be treated and whether under certain circumstances long-term prophylaxis is necessary. At this regard, systemic comorbidities, immune-genetic status, laterality, residual vision, and history of recurrences should be considered. Although delayed onset ARN is rare, physicians must consider the possibility of the development of ARN in the fellow eye even many years after the initial onset. In current practice, patients are often given intravitreal injections of foscarnet or ganciclovir to provide high-dose immediate treatment to the eye and attain immediate therapeutic vitreous drug levels and inhibition of viral replication. There is a strong scientific rationale to support the adjunctive use of intravitreal foscarnet (2.4 mg) in the early treatment of ARN in adults to reduce the risk of severe vision loss and incidence of RD (1). Intravitreal injections of foscarnet and ganciclovir were reported in 7 and 11 patients, respectively. The doses used ranged from 1.2 to 2.4 mg for foscarnet and was 2 mg/0.05 ml for ganciclovir. No side effects were reported. Early pars plana vitrectomy and/or argon laser photocoagulation have been proposed to relieve vitreoretinal traction and try to prevent the risk of RD; however, the benefit of these procedures remains controversial (1).

Visual prognosis is variable but usually poor. There is no general agreement about factors influencing visual improvement; however, low initial BCVA, advanced clinical picture, retinal detachment, and VZV-related etiology have been often associated with poor prognosis in the general population (11, 29, 30). In accordance with previous studies, we found a significant correlation between BCVA worsening and retinal detachment. Among pediatric patients, lower ages are associated with better outcomes; of note, our analysis excluded neonatal ARN as visual acuity could not be performed, thus introducing a selection bias.

Systemic corticosteroids (usually prednisone up to 1 mg/kg/die) are often co-administered with antiviral therapy to suppress intraocular inflammation and improve vitreous opacification. The latter may explain the association we found between steroids and BCVA improvement. In clinical practice, treatment with corticosteroids is begun 48–72 h after initiation of antiviral therapy, to reduce the risk of promoting viral replication (31).

**Figure 4:**
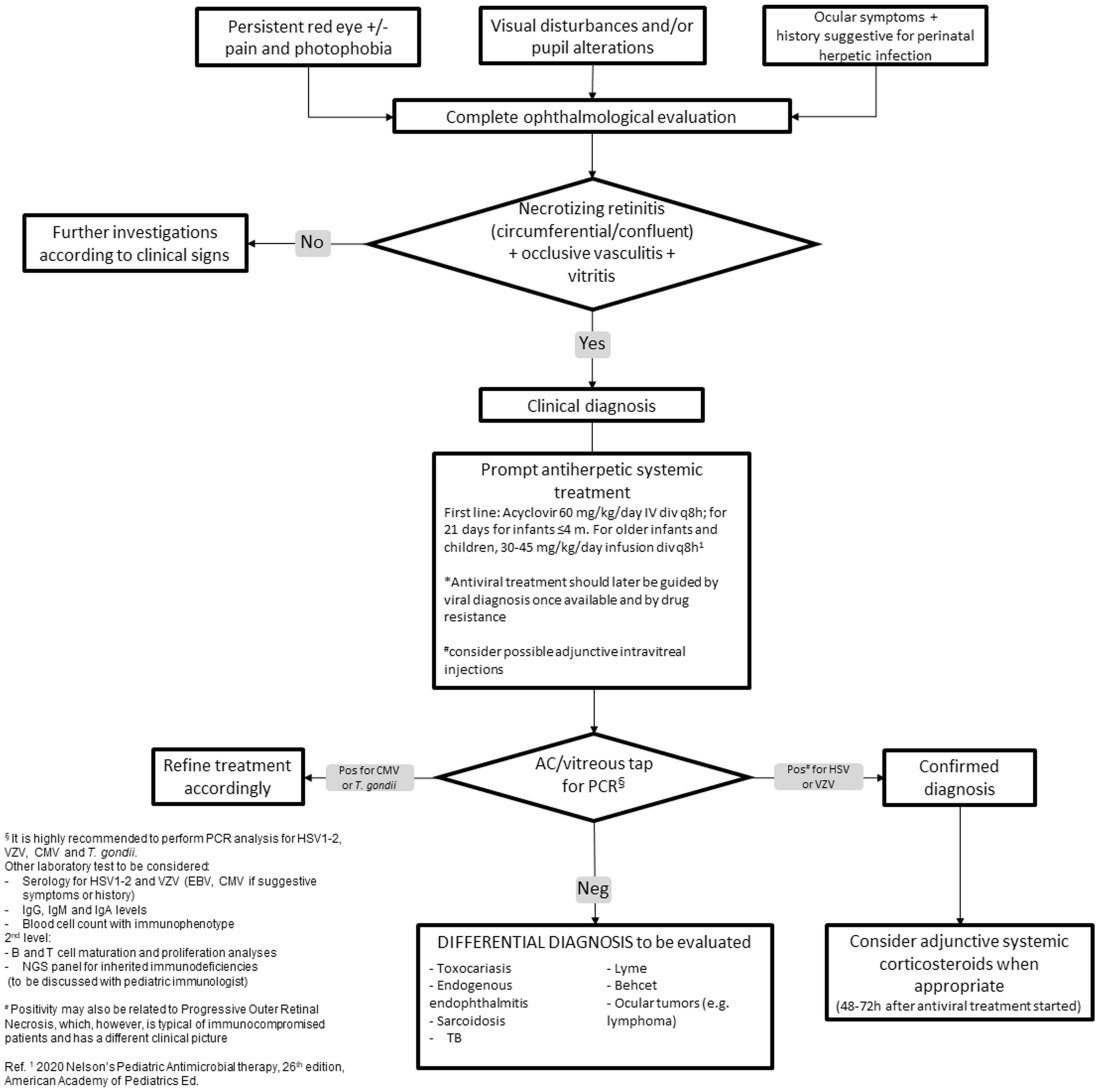
Flow chart summarizing the recommended approach to the patient with suspicion of acute retinal necrosis (ARN). PCR: protein chain reaction; AC: anterior chamber; CMV: cytomegalovirus; HSV1–2: herpes simplex virus 1–2; VZV: varicella-zoster virus; EBV: Epstein–Barr virus; PORN: progressive outer retinal necrosis; TB: tuberculosis.

Management of uveitis in children can be more challenging then in adults. The inability to verbalize the complaint could lead to delays in diagnosing both primary disease and later complications; moreover, a complete eye examination can be arduous due to poor cooperation. However, a timely diagnosis and treatment, together with careful monitoring of sight-threatening complications, are crucial in ARN to guarantee the best visual prognosis achievable and prevent lifelong disabilities, especially in pediatric population whose visual system is still immature with the specific risk of developing amblyopia. For these reasons, we propose a simple approach to the diagnosis and management of pediatric patients with suspected ARN in Figure 4.

The main limitation of this analysis is the heterogeneity of the collected data as no natural history studies or prospective clinical trials on ARN in pediatric patients are currently available. However, this is the first study that deals with the topic, attempting a better definition of the disease management and the identification of clinical prognostic factors.

In conclusion, ARN in children is a rare disease, associated with a potential poor prognosis if not promptly and aggressively treated. Therefore, in the pediatric population, a high index of suspicion is particularly crucial to achieve a prompt diagnosis and appropriate treatment.

## Data Availability Statement

The raw data supporting the conclusions of this article will be made available by the authors, without undue reservation.

## Ethics Statement

Written informed consent was obtained from the individual(s), and minor(s)' legal guardian/next of kin, for the publication of any potentially identifiable images or data included in this article.

## Author Contributions

CM and GF: conceived the idea, conceptualized the study, and drafted the manuscript. PM and CD: collected the data. MN, CD, and PM: analyzed the data. FM, SO, FV, CA, and GF: reviewed the manuscript. All authors read and approved the final manuscript. All authors contributed to the article and approved the submitted version.

## Funding

Publication [The APC] costs were funded by Grant Ricerca Corrente, Italian Ministry of Health.

## Conflict of Interest

The authors declare that the research was conducted in the absence of any commercial or financial relationships that could be construed as a potential conflict of interest.

## Publisher's Note

All claims expressed in this article are solely those of the authors and do not necessarily represent those of their affiliated organizations, or those of the publisher, the editors and the reviewers. Any product that may be evaluated in this article, or claim that may be made by its manufacturer, is not guaranteed or endorsed by the publisher.
